# Using the canine microbiome to bridge translation of cancer immunotherapy from pre-clinical murine models to human clinical trials

**DOI:** 10.3389/fimmu.2022.983344

**Published:** 2022-08-12

**Authors:** Kara T. Kleber, Khurshid R. Iranpur, Lauren M. Perry, Sylvia M. Cruz, Aryana M. Razmara, William T. N. Culp, Michael S. Kent, Jonathan A. Eisen, Robert B. Rebhun, Robert J. Canter

**Affiliations:** ^1^ Division of Surgical Oncology, Department of Surgery, University of California Davis Medical Center, Sacramento, CA, United States; ^2^ School of Veterinary Medicine, University of California Davis, Sacramento, CA, United States; ^3^ Center for Companion Animal Health Department of Surgical and Radiological Sciences, School of Veterinary Medicine, University of California Davis, Davis, CA, United States; ^4^ Department of Medical Microbiology and Immunology, University of California Davis, Davis, CA, United States

**Keywords:** microbiome, immunotherapy, cancer, comparative oncology, canines

## Abstract

The microbiome has clearly been established as a cutting-edge field in tumor immunology and immunotherapy. Growing evidence supports the role of the microbiome in immune surveillance, self-tolerance, and response to immune checkpoint inhibitors such as anti PD-L1 and CTLA-4 blockade (1–6). Moreover, recent studies including those using fecal microbial transplantation (FMT) have demonstrated that response to checkpoint immunotherapies may be conferred or eliminated through gut microbiome modulation (7, 8). Consequently, studies evaluating microbiota-host immune and metabolic interactions remain an area of high impact research. While observations in murine models have highlighted the importance of the microbiome in response to therapy, we lack sufficient understanding of the exact mechanisms underlying these interactions. Furthermore, mouse and human gut microbiome composition may be too dissimilar for discovery of all relevant gut microbial biomarkers. Multiple cancers in dogs, including lymphoma, high grade gliomas, melanomas and osteosarcoma (OSA) closely resemble their human analogues, particularly in regard to metastasis, disease recurrence and response to treatment. Importantly, dogs with these spontaneous cancers also have intact immune systems, suggesting that microbiome analyses in these subjects may provide high yield information, especially in the setting of novel immunotherapy regimens which are currently expanding rapidly in canine comparative oncology (9, 10). Additionally, as onco-microbiotic therapies are developed to modify gut microbiomes for maximal responsiveness, large animal models with intact immune systems will be useful for trialing interventions and monitoring adverse events. Together, pre-clinical mechanistic studies and large animal trials can help fully unlock the potential of the microbiome as a diagnostic and therapeutic target in cancer.

## Success and limitations of the murine model

Murine models represent the most used model for studying host-microbiome physiology at both functional and mechanistic levels. Due to genetic homogeneity and laboratory environments, mouse models allow for high levels of control and improved experimental reproducibility (11). In cancers with representative murine models, mice have uncovered cancer promoting microbiota, and microbiota associated with improved response to treatment. Murine colon cancer models, for example, have contributed to understanding the influence of the gut microbiome on colorectal tumorigenesis *via* modulation of inflammation. In these models, transfer of the microbiome from tumor-bearing mice to germ free mice accelerated tumor growth, demonstrating causality (12, 13). Similarly, TLR4 knockout mice have been critical in understanding the oncogenic effect of gut microbiota in liver cancer (14). Sivan et al (15)demonstrated key differences in tumor kinetics and responses to immunotherapy between genetically similar mice bred in two different environments. One cohort showed anti-tumor effects following anti-PD1 checkpoint inhibitor therapy, while the other cohort showed no benefit. However, co-housing the mice together reversed the differences in anti-tumor responses, which were then restored by the administration of oral Bifidobacterium spp (15). Similar observations were made for mice receiving anti-CTLA checkpoint inhibitors with effects modulated by Bacteroides species, (specifically *B. fragilis* and/or *B. thetaiotaomicrom*) (16).

Despite the strength of these mechanistic murine studies, there are limitations to mouse models, particularly when considering the complex interactions between microbiome, immune response, and cancer. Laboratory mice demonstrate different microbial compositions to their wild counterparts. Attempts to convert lab-type to wild type mice have successfully altered, but not completely recapitulated, natural microbiome profiles (17). Moreover, animal facilities themselves significantly impact microbiome composition. For example, key differences in dominant taxa have been observed between identical mouse lines from different animal locations (18). Similarly, the use of germ-free mice is both a strength and a weakness of mouse models (19). These sterile conditions allow mice to be born without microbial colonization prior to the introduction of species of interest. However, this germ-free environment also leads to significant impairment of immune system development and responses. For example, mice raised in sterile conditions develop higher levels of IgE and fail to induce the same levels of immune reactivity seen during the “weaning period” of wild-type mice (20–23). This results in susceptibility to certain bacteria and increased risk of immunopathologies, complications which are not resolved with instillation of an “adult” or “wild-type” microbiome (21, 22, 24). Additionally, mouse studies of the microbiome show a large genetic influence which is distinct from humans where <10% of taxa are thought to be heritable (24–28). The high level of genetic homogeneity present in most murine models raises questions of generalizability to the human situation where genetic backgrounds and environmental exposures are vastly more diverse. This concern is reinforced by studies which have demonstrated the impact of environmental influences especially diet and drugs, as the most prominent factors influencing the microbiome in humans (29–31).

Additionally, as laboratory animals, mice do not receive the multi-faceted cancer care that is provided to humans and companion animals such as dogs. While pre-clinical murine models of checkpoint blockade immunotherapy have demonstrated impressive ability to prevent disease progression in various cancer cell lines, translation to human medicine has shown mixed results (32). Clinically meaningful survival benefits of PD-1 & CTLA-4 checkpoint inhibitor therapy have been demonstrated in some cancers, such as melanoma and non-small cell lung cancer, but only in a subset of patients (31–34)While observations from murine studies have demonstrated microbiome-associated factors for responsiveness to treatment, another limitation of murine models is their tendency to be reductionistic in their capacity to explore all potential intersecting variables which influence the cancer-immunity cycle (5–8, 15, 35, 36).

Similarly, murine models are able to strictly control diet and environment, both of which are key drivers of microbiome plasticity and composition, but as with germ-free studies, this can be both a strength and a weakness. For example, Matson et al. and Gopalakrishnan et al. (5, 6) used FMT to study whether human commensal microbes would potentiate anti-tumor T cell responses in germ-free mice in two separate studies. Matson et al. demonstrated slower tumor growth in some mice after transferring microbiota from human responders. Gopalakrishnan et al. (6) showed changes in tumor infiltrating cells and upregulation of PD-1 in the tumor microenvironment after FMT. Although these mouse experiments demonstrated significant proof-of-concept results, it is not clear why effects were seen in some, but not all, mice. Given that the mice were evaluated to be highly homogenous and genetically inbred, the mice are expected to respond similarly. It is unclear whether the effects on the treatment would persist when using models with more heterogenous genetic backgrounds and environmental exposures. Additionally, the fact that similar mechanisms were identified in these studies but the precise microorganisms were different highlights potential concerns regarding generalizability and extrapolation as noted above.

Microbiome studies in dogs with spontaneous cancers receiving immunotherapy offer an additional avenue for exploration. As the development of canine immunotherapy advances, including the development of novel caninized anti-PD1, anti-CTLA-4 and anti-PD-L1 monoclonal antibodies, microbiome correlative studies and attempted therapeutic intervention in canines with intact immune systems and a lifetime of commensal gut microbiome symbionts may yield useful observations for human applications (37–39). Human clinical immunotherapy trials are impacted by immune-related adverse effects such as colitis and myocarditis (32, 33, 39, 40). While murine models have been developed to help study these immune-related events, these models are prone to develop auto-immune responses which may limit translational potential (41). Canine models represent an opportunity to bridge mechanistic studies in mice with descriptive studies in humans, and microbiome studies in companion animal dogs can help advance our understanding of how the gut microbiome shapes immunotherapy responses as well as toxicity and adverse events.

## Human vs canine microbiome

As in humans, studies of the canine microbiome have evaluated the relationship of the microbiome with inflammatory disease states, development of malignancies, and more recently, response to oncologic therapies (42–47). The “normal” adult microbiota comprises thousands of bacterial species across mucosal and skin surfaces and is determined by environmental and genetic factors. The colonization of both the human and canine gut microbiome, one of the densest bacterial environments on earth, increases in alpha diversity and decreases in beta diversity during the weaning period before stabilizing (0-3 years in humans, 0-9 weeks in canines) (45, 48–50). Exposure to microbes during delivery, early diet, and antibiotic exposure have all been shown to impact gut microbial development (22, 51, 52). Early influences have clinical impact – colonization with *C. difficile* during the first months of life is associated with increased risk of atopic disorders such as asthma and eczema in children (53). Microbiome compositions in pre-weened puppies display instability of prominent taxa. Puppies that displayed these “immature” microbiome profiles are more suspectilble to diarrheal illness compared to those who have developed stable more “adult” compositions (54). Environmental factors such as diet, drugs, and living conditions also exert key influences on subsequent adult microbiota composition (29, 30). Microbiomes of genetically unrelated co-habitants are consistently demonstrated to be more similar than those of relatives who do not cohabitate, and genetic ancestry does not predict compositional similarities (29, 55). While environment is the primary influencer of composition, there appears to be heritability in human and canine gut microbiomes as well. A study of 400 human twin pairs raised in the same household compared monozygotic vs dizygotic siblings. Interestingly, monozygotic twins demonstrated more similar gut microbiota compared to their dizygotic counterparts (56). Canine comparisons of dog breeds show similar findings, although studies of breed variation indicate that breed does not cause major shifts in microbiome composition or diversity but may influence abundance of specific taxa (46). Although not to the extent of humans or mice, the canine gut microbiota has been surveyed in some detail (50, 57–59). Most large studies agree on the five most prominent phyla in dogs: *Firmicutes, Fusobacteria, Bacteroides, Proteobacteria and Actinobacteria*, the composition of which is more similar to humans than other commonly studied mammalian species. Coelho et al. (57) compared 129 stool samples from 64 dogs against previously published gut microbial gene catalogs based on similar sequencing methods. The phylum level distribution of genes in the dog was more like the published human data than that of a mouse or pig. When genes were clustered by each species pool, the dog gut gene pool overlapped most with the human gut microbiome (23%) compared to only 4.9% for the murine gene catalog. As in prior human data, the majority of the composition appeared to be influenced by environmental exposures, diet, and disease states. Importantly, certain bacterial species have been found to exert similar effect across species. Obesity, which is a growing problem in both humans and canines, is correlated with a change in Firmicutes/Bacteroides ratios in both humans and canines (60–62). Canine and human inflammatory bowel disorders are associated with a reduction in microbial community diversity and structure characterized by loss of key species or overgrowth of species with genotoxic potential such as *Bacteroides fragilis* and *E. coli* (45, 63). In humans, these microbiome shifts have been associated with colorectal cancer (12, 64, 65). While colorectal cancer is less common in dogs, increased *E.coli* is associated with canine intestinal lymphoma (42). Importantly, human and dog differences in microbiome interactions are also informative. Fusobacterium, for instance, plays a role in energy utilization in the gut as a fermenting species. In humans, *F. nucleatum* is associated with colorectal cancer and thought to trigger inhibitory T cell receptors that suppress anti-cancer immune response (66, 67). In dogs, however, fusobacteria are associated with maintaining gut health (67).

While similarities in the development and composition of the gut microbiome present one advantage of the canine model, perhaps the most important is that of a shared environment with humans. As companion animals, dogs inhabit the same environment as humans. Therefore, shifts in composition caused by disease states or therapeutics must be robust enough to compete with the myriad of “real life” influences that patients encounter. Shared environments may also lead to shared taxonomy; canine housemates show similar microbiome profiles, similar to what is observed between human cohabiting with a spouse or partner (68). Interestingly, co-living between species influences microbiome profile as well (69). Companion dogs share more skin flora with their owners than with other dogs and oral microbiomes, which may show relevance in oral melanoma, as there is evidence of oral spread of symbionts between humans and dogs (68, 69). Some have hypothesized that pet ownership may influence the human gut microbiome as well (70). Small differences in abundance for two OTUs have been associated with pet ownership, although additional studies are needed given the risk of type I error (69).

## Microbiome in canine therapeutics

Attenuation of immune surveillance and development of immunological tolerance to tumor-derived antigens contribute to cancer development and progression. Novel immunotherapies target one of these two mechanisms to stimulate the subject’s immune system to recognize and target cancer as non-self. Overcoming both intrinsic and acquired resistance to these treatments are critical areas of study, and although several species of interest have been identified, no universal “responder signature” has been identified. Canine analysis that identifies similar bacterial enrichment as in human studies would greatly enhance the chances of replicable and generalizable effects.

Microbiome modulation has demonstrated exciting promise in increasing the number of human patients who may respond to PD-1/PD-L1 checkpoint inhibitor immunotherapy (4–6). Demonstrated first by Gopalakrishnan & Matson et al. (5, 6) in a murine model, and recently by Baruch et al. (7) in a human clinical trial, FMT from a drug-responsive subject to a nonresponsive subject was able to convert a subset of prior non-responders, allowing them to benefit from treatment. Importantly, when considering the human trial of FMT, extensive pre-and post-treatment analysis regarding microbiome composition, local metabolism, and expression of immune-related genes failed to show significant differences between those who became responsive to treatment and those who did not (7). Thus, while successful in proving the concept of FMT to be safe and potentially beneficial, the effects remain correlative. These types of immune-microbiome relationships could benefit from additional studies in dogs with spontaneous cancers in the setting of an intact immune system. FMT is accepted in community veterinary practice making high-powered studies possible once commercially available caninized ICI’s are readily available (59). Repeating FMT trials with canine immunotherapy provides an opportunity to integrate observations of gut microbial alterations with longitudinal changes in metabolism and immune surveillance. Dogs, unlike mice, allow for multiple collections of adequate blood samples that can detect metabolites of short chain fatty acids that can act as ligands for G-protein coupled receptors. Iosine and hypoxanthine have recently been associated with bacteria of interest, for example (71, 72).

While veterinary medicine is waiting for the widespread commercial availability of canine specific PD-1/PD-L1/CTLA-4 mAbs to usher the area of immunotherapy in dogs, other immunotherapy approaches are in use, and comparison of microbiome profiles between responder status in other immune-based therapies should be considered (73, 74). Longitudinal stool and blood sampling of these canine patients for microbiome and metabolic investigation presents a rich area of investigation. Standard treatments for dog cancers include chemotherapy and radiation, which may allow for evaluation of the impacts that traditional therapies have on the microbiome alone or in combination with immunotherapy. Canine lifecycles are also inherently shorter, so rapidly progressing canine cancers allow for collection of samples linked to critical end points such as disease-free interval and time to progression which are highly relevant to the human situation.

## Microbiome in the tumor microenvironment

The gut microbiome represents the most extensively classified community in humans and animal models, yet increasing studies have demonstrated that microbial communities in other compartments have clinical implications (74–76). Tumor-associated microbiomes have been identified in multiple human cancers including breast, lung, pancreas, and melanoma tumors (74–79). While tumor infiltrating lymphocytes (TILs) have emerged as a key feature of the immune tumor microenvironment (TME), a deeper molecular and cellular signals responsible for immune-tumor-microbiome communication are not yet understood. While some studies demonstrate a higher number of tumor infiltrating lymphocytes (TILs) in the TME project more positive patient outcomes, others suggest that the upregulation of regulatory T cells in the TME is associated with a more negative outcome (80–82). Although mouse models can provide key insights into the immune-tumor interactions in the TME and are important for mechanistic studies, these models have limitations given the nature of transplanted highly retroviral tumors. Known differences in tumor initiation and promotion are likely to impact the fidelity of the model for microbiome studies (83, 84).

Therefore, dogs represent an important translational opportunity for studies of the cancer-immunity cycle, including microbiome studies. Canine immunotherapy, including evaluation of novel therapies in the context of clinical trials, has received significant attention from researchers and funding agencies, including the Cancer Moonshot, and examples of ongoing canine immunotherapy trials can be seen in **Table 1** (9, 10). Dogs are diagnosed with naturally occurring cancers which are highly similar to humans, and genetic studies have demonstrated notable overlap in the tumor genetic makeup of human and dog OSA, melanoma, mammary tumors, gliomas and lymphoma (84–90). Canine OSA recapitulates several feature of human OSA including frequent TP53, PI3K, and MAPK pathway mutations with low expression of immuno-associated genes and a trend toward higher mutation burden in metastatic disease (88). Canine mammary tumors and lymphomas share major gene alterations with their human counterparts in addition to having similar tumor mutation burdens (91). Gliomas have been shown to share specific alterations in receptor tyrosine kinases such as TP53 and cell cycle pathways with human pediatric gliomas (89). Overall, the incidence of these canine cancers is believed to be as high or higher than their human counterparts, although precise case numbers are not known since no national database exists for dogs. For example, soft tissue sarcomas make up 10-15% of malignant tumors in dogs with estimates of approximately 7700-31,800 new canine cases per year in the United States compared to soft tissue sarcoma in humans which represents approximately 1% of cancer cases and 10,000 – 12,000 new cases per year in the US (91). Similarly, canine OSA is estimated at approximately 25,000 – 50,000 cases per year, whereas the number of human OSA is approximately 3000 per year in the US (92–94). Data such as these highlight the value of the canine model as an important asset in tumor immunology and immunotherapy studies, especially where an intact immune system is critical for understanding tumor and host biology.

**Table 1:** Ongoing Clinical Trials in Dogs.

Institution	Clinical Trial	Cancer Type
University of California Davis	Enhancing Natural Killer Immunotherapy with First in Dog Trials of Inhaled Recombinant IL-15 and Super agonist IL-15 in Naturally Occurring Canine Cancers	Osteosarcoma Melanoma
University of Alabama at Birmingham	Canine Immuno-Neurotherapeutics (Combination immunotherapies for canine brain tumors)	Glioma
Colorado State University	Optimizing Novel Immunotherapy Combination Targeting the Tumor Microenvironment in Canine Spontaneous Osteosarcoma	Osteosarcoma
Tufts University	Enhancing the Efficacy of Immunotherapy in DLBCL Using Rational Combination Approaches	Lymphoma
University of Minnesota	Novel Combined Immunotherapeutic Strategies for Glioma: Using Pet Dogs as a Large Animal Spontaneous Model	Glioma

## Conclusion

Murine models will always have utility as pre-clinical models, including for microbiome studies. However, due to the complex interactions between the immune system, the TME, and metabolism, utilization of canines for microbiome research is a promising strategy to yield additional data that can bridge our understanding from “proof of concept” to “proof of mechanism”. Further classification of the canine microbiome in cancer, changes in the canine microbiome in response to immunotherapy, and characterization of microbiomes outside the gut will all be important in deriving the most from canine models. This is particularly important now as veterinary medicine has made advances in canine specific immunotherapy agents that mirror human ICI. Canine clinical trials should consider collection of stool samples for microbiome biomarkers, as well as tissue and blood samples as well to correlate microbiome changes with immune infiltration and metabolic changes. As we increase our understanding of the interplay between specific commensal bacteria and the targets of immune therapy, the next step is therapeutic application. Through diet, antibiotics, fecal transfer, or other methods, reliably tailoring gut microbiomes to create maximal responsiveness with minimal side effects is the future of immuno-oncology. Dogs present a step-in bench to bedside science where their developing interventions can be tested on immune intact models exposed to complex environmental influences to ensure they demonstrate durability and utility.

## Author contributions

KK and RC conceptualized the manuscript. KK, KI, LP, SC, and AR reviewed current literature. KK and KI wrote the manuscript and generated the associate figure. WC, MK, JE, and RR edited the manuscript and provided context for veterinary literature. RC edited the final paper. All authors contributed to the article and approved the submitted version.

## Funding

This work was supported in part by National Institutes of Health/National Cancer Institute grants U01 CA224166-01 (RC and RR), R03CA252793 (RC), and T32CA251007 (KK, LP, MK, and RC).

## Acknowledgments

**Figure 1:**
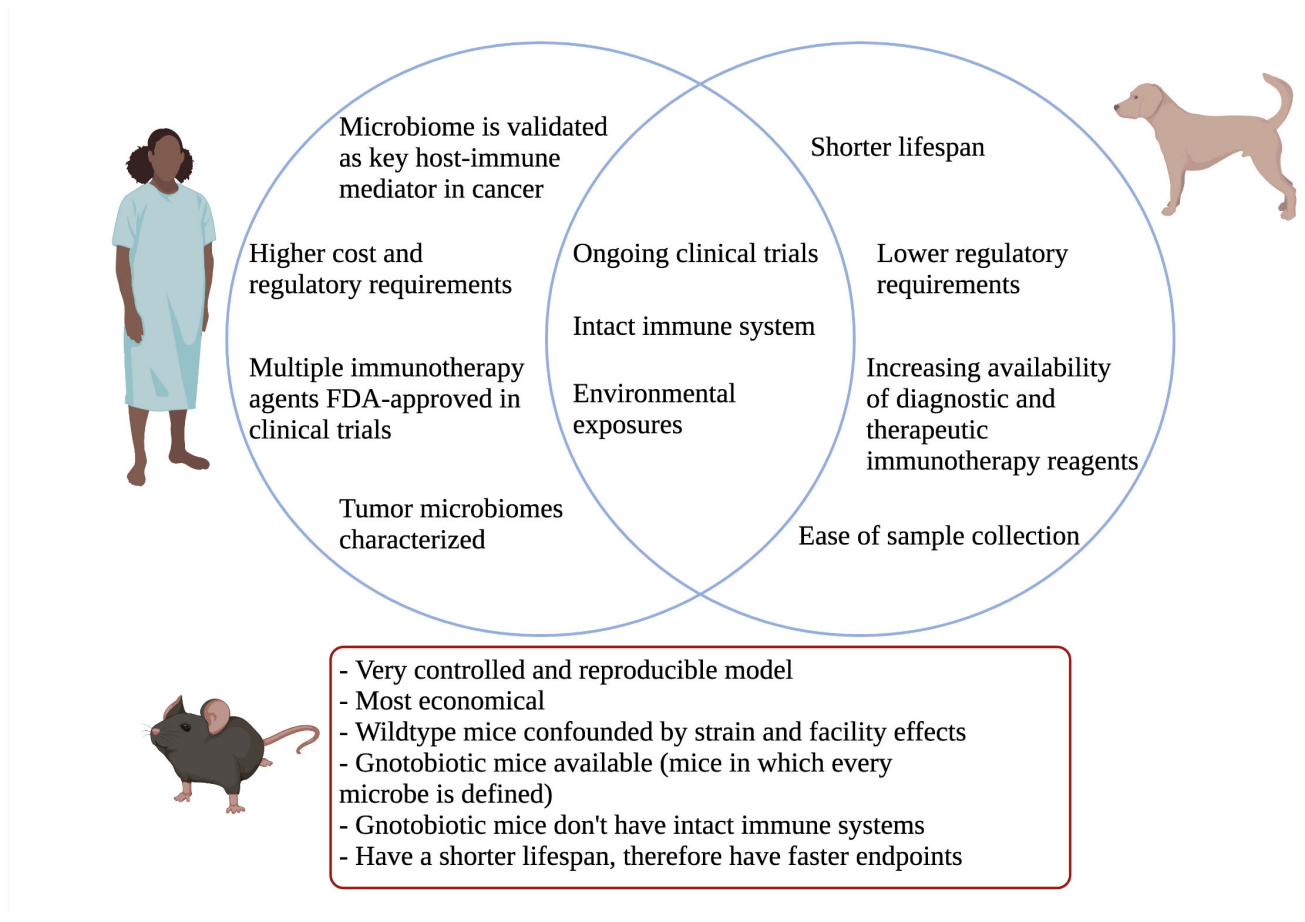
Venn diagram comparing relevant aspects of canine model to humans. Important mouse model differences highlighted below.

We would like to thank the members of the Laboratory for Cancer Immunology at UC Davis for their scientific and editorial input. **Figure 1** was created with Biorender.com.

## Conflict of interest

The authors declare that the research was conducted in the absence of any commercial or financial relationships that could be construed as a potential conflict of interest.

## Publisher’s note

All claims expressed in this article are solely those of the authors and do not necessarily represent those of their affiliated organizations, or those of the publisher, the editors and the reviewers. Any product that may be evaluated in this article, or claim that may be made by its manufacturer, is not guaranteed or endorsed by the publisher.

## References

[1] Frank DN St Amand AL Feldman RA Boedeker EC Harpaz N Pace NR. Molecular-phylogenetic characterization of microbial community imbalances in human inflammatory bowel diseases. Proc Natl Acad Sci USA (2007) 104(34):13780–5. doi: 10.1073/pnas.0706625104 PMC1959459 17699621

[2] Rodrigues Hoffmann A. The cutaneous ecosystem: the roles of the skin microbiome in health and its association with inflammatory skin conditions in humans and animals. Vet Dermatol (2017) 28(1):60–e15. doi: 10.1111/vde.12408 28133874

[3] Ni J Shen T-CD Chen EZ Bittinger K Bailey A Roggiani M. A role for bacterial urease in gut dysbiosis and crohn’s disease. Sci Transl Med (2017) 9(416):1–11. doi: 10.1126/scitranslmed.aah6888 PMC5808452 29141885

[4] Routy B Le Chatelier E Derosa L Duong CPM Alou MT Daillère R. Gut microbiome influences efficacy of PD-1-based immunotherapy against epithelial tumors. Science (2018) 359(6371):91–7. doi: 10.1126/science.aan3706 29097494

[5] Matson V Fessler J Bao R Chongsuwat T Zha Y Alegre M-L. The commensal microbiome is associated with anti–PD-1 efficacy in metastatic melanoma patients. Science (2018) 359. 10.1126/science.aao3290 PMC6707353 29302014

[6] Gopalakrishnan V Spencer CN Nezi L Reuben A Andrews MC Karpinets TV. Gut microbiome modulates response to anti-PD-1 immunotherapy in melanoma patients. Science (2018) 359(6371):97–103. doi: 10.1126/science.aan4236 29097493 PMC5827966

[7] Baruch EN Youngster I Ben-Betzalel G Ortenberg R Lahat A Katz L. Fecal microbiota transplant promotes response in immunotherapy-refractory melanoma patients. Science (2021) 371(6529):602–9. doi: 10.1126/science.abb5920 33303685

[8] Davar D Dzutsev AK McCulloch JA Rodrigues RR Chauvin J-M Morrison RM. Fecal microbiota transplant overcomes resistance to anti-PD-1 therapy in melanoma patients. Science (2021) 371(6529):595–602. doi: 10.1126/science.abf3363 33542131 PMC8097968

[9] Park JS Withers SS Modiano JF Kent MS Chen M Luna JI. Canine cancer immunotherapy studies: linking mouse and human. J Immunother Cancer (2016) 4:97. doi: 10.1186/s40425-016-0200-7 28031824 PMC5171656

[10] LeBlanc AK Mazcko CN. Improving human cancer therapy through the evaluation of pet dogs. Nat Rev Cancer (2020) 20(12):727–42. doi: 10.1038/s41568-020-0297-3 32934365

[11] Nguyen TLA Vieira-Silva S Liston A Raes J. How informative is the mouse for human gut microbiota research? Dis Model Mech (2015) 8(1):1–16. doi: 10.1242/dmm.017400 25561744 PMC4283646

[12] Zackular JP Baxter NT Iverson KD Sadler WD Petrosino JF Chen GY. The gut microbiome modulates colon tumorigenesis. MBio (2013) 4(6):e00692–13. doi: 10.1128/mBio.00692-13 PMC3892781 24194538

[13] Grivennikov SI Wang K Mucida D Stewart CA Schnabl B Jauch D. Adenoma-linked barrier defects and microbial products drive IL-23/IL-17-mediated tumour growth. Nature (2012) 491(7423):254–8. doi: 10.1038/nature11465 PMC3601659 23034650

[14] Dapito DH Mencin A Gwak G-Y Pradere J-P Jang M-K Mederacke I. Promotion of hepatocellular carcinoma by the intestinal microbiota and TLR4. Cancer Cell (2012) 21(4):504–16. doi: 10.1016/j.ccr.2012.02.007 PMC3332000 22516259

[15] Sivan A Corrales L Hubert N Williams JB Aquino-Michaels K Earley ZM. Commensal bifidobacterium promotes antitumor immunity and facilitates anti-PD-L1 efficacy. Science (2015) 350(6264):1084–9. doi: 10.1126/science.aac4255 PMC4873287 26541606

[16] Vétizou M Pitt JM Daillère R Lepage P Waldschmitt N Flament C. Anticancer immunotherapy by CTLA-4 blockade relies on the gut microbiota. Science (2015) 350(6264):1079–84. doi: 10.1126/science.aad1329 PMC4721659 26541610

[17] Rosshart SP Herz J Vassallo BG Hunter A Wall MK Badger JH. Laboratory mice born to wild mice have natural microbiota and model human immune responses. Science (2019) 365(6452):eaaw4361. doi: 10.1126/science.aaw4361 31371577 PMC7377314

[18] Parker KD Albeke SE Gigley JP Goldstein AM Ward NL. Microbiome composition in both wild-type and disease model mice is heavily influenced by mouse facility. Front Microbiol (2018) 9:1598. doi: 10.3389/fmicb.2018.01598 30079054 PMC6062620

[19] Cahenzli J Köller Y Wyss M Geuking MB McCoy KD. Intestinal microbial diversity during early-life colonization shapes long-term IgE levels. Cell Host Microbe (2013) 14(5):559–70. doi: 10.1016/j.chom.2013.10.004 PMC4049278 24237701

[20] Al Nabhani Z Dulauroy S Marques R Cousu C Al Bounny S Déjardin F. A weaning reaction to microbiota is required for resistance to immunopathologies in the adult. Immunity (2019) 50(5):1276–1288.e5. doi: 10.1016/j.immuni.2019.02.014 30902637

[21] Lubin J-B Green J Maddux S Denu L Duranova T Lanza M. Arresting microbiome development limits immune system maturation and resistance to infection. bioRxiv (2022) 1. doi: 10.1101/2022.01.17.476513v1.abstract PMC10935632 36996818

[22] Bayer F Ascher S Pontarollo G Reinhardt C. Antibiotic treatment protocols and germ-free mouse models in vascular research. Front Immunol (2019) 10:2174. doi: 10.3389/fimmu.2019.02174 31572384 PMC6751252

[23] Labuda JC Fong KD McSorley SJ. Cohousing with dirty mice increases the frequency of memory T cells and has variable effects on intracellular bacterial infection. Immunohorizons (2022) 6(2):184–90. doi: 10.4049/immunohorizons.2100069 PMC9624231 35210292

[24] Korach-Rechtman H Freilich S Gerassy-Vainberg S Buhnik-Rosenblau K Danin-Poleg Y Bar H. Murine genetic background has a stronger impact on the composition of the gut microbiota than maternal inoculation or exposure to unlike exogenous microbiota. Appl Environ Microbiol (2019) 85(18). doi: 10.1128/AEM.00826-19 PMC6715835 31350316

[25] Bubier JA Chesler EJ Weinstock GM. Host genetic control of gut microbiome composition. Mamm Genome (2021) 32(4):263–81. doi: 10.1007/s00335-021-09884-2 PMC8295090 34159422

[26] Campbell JH Foster CM Vishnivetskaya T Campbell AG Yang ZK Wymore A. Host genetic and environmental effects on mouse intestinal microbiota. ISME J (2012) 6(11):2033–44. doi: 10.1038/ismej.2012.54 PMC3475380 22695862

[27] Org E Parks BW Joo JWJ Emert B Schwartzman W Kang EY. Genetic and environmental control of host-gut microbiota interactions. Genome Res (2015) 25(10):1558–69. doi: 10.1101/gr.194118.115 PMC4579341 26260972

[28] Gacesa R Kurilshikov A Vich Vila A Sinha T Klaassen MAY Bolte LA. Environmental factors shaping the gut microbiome in a Dutch population. Nature (2022) 604(7907):732–9. doi: 10.1038/s41586-022-04567-7 35418674

[29] David LA Maurice CF Carmody RN Gootenberg DB Button JE Wolfe BE. Diet rapidly and reproducibly alters the human gut microbiome. Nature (2014) 505(7484):559–63. doi: 10.1038/nature12820 PMC3957428 24336217

[30] Simões CD Maukonen J Kaprio J Rissanen A Pietiläinen KH Saarela M. Habitual dietary intake is associated with stool microbiota composition in monozygotic twins. J Nutr (2013) 143(4):417–23. doi: 10.3945/jn.112.166322 23343669

[31] Chae YK Arya A Iams W Cruz MR Chandra S Choi J. Current landscape and future of dual anti-CTLA4 and PD-1/PD-L1 blockade immunotherapy in cancer; lessons learned from clinical trials with melanoma and non-small cell lung cancer (NSCLC). J Immunother Cancer (2018) 6(1):39. doi: 10.1186/s40425-018-0349-3 29769148 PMC5956851

[32] Lynch TJ Bondarenko I Luft A Serwatowski P Barlesi F Chacko R. Ipilimumab in combination with paclitaxel and carboplatin as first-line treatment in stage IIIB/IV non–Small-Cell lung cancer: Results from a randomized, double-blind, multicenter phase II study. J Clin Orthod (2012) 30(17):2046–54. doi: 10.1200/JCO.2011.38.4032 22547592

[33] Hodi FS O’Day SJ McDermott DF Weber RW Sosman JA Haanen JB. Improved survival with ipilimumab in patients with metastatic melanoma. N Engl J Med (2010) 363(8):711–23. doi: 10.1056/NEJMoa1003466 PMC3549297 20525992

[34] Haslam A Gill J Prasad V. Estimation of the percentage of US patients with cancer who are eligible for immune checkpoint inhibitor drugs. JAMA Netw Open (2020) 3(3):e200423. doi: 10.1001/jamanetworkopen.2020.0423 32150268 PMC7063495

[35] Choi JW Withers SS Chang H Spanier JA de la Trinidad VL Panesar H. Development of canine PD-1/PD-L1 specific monoclonal antibodies and amplification of canine T cell function. PloS One (2020) 15(7):e0235518. doi: 10.1371/journal.pone.0235518 32614928 PMC7332054

[36] Rothschild D Weissbrod O Barkan E Kurilshikov A Korem T Zeevi D. Environment dominates over host genetics in shaping human gut microbiota. Nature (2018) 555(7695):210–5. doi: 10.1038/nature25973 29489753

[37] Mason NJ Chester N Xiong A Rotolo A Wu Y Yoshimoto S. Development of a fully canine anti-canine CTLA4 monoclonal antibody for comparative translational research in dogs with spontaneous tumors. MAbs (2021) 13(1):2004638. doi: 10.1080/19420862.2021.2004638 34856888 PMC8726733

[38] Marable J Ruiz D Jaiswal AK Bhattacharya R Pantazes R Agarwal P. Nanobody-based CTLA4 inhibitors for immune checkpoint blockade therapy of canine cancer patients. Sci Rep (2021) 11(1):20763. doi: 10.1038/s41598-021-00325-3 34675296 PMC8531395

[39] Yang JC Beck KE Blansfield JA Tran KQ Lowy I Rosenberg SA. Tumor regression in patients with metastatic renal cancer treated with a monoclonal antibody to CTLA4 (MDX-010). J Clin Orthod (2005) 23(16_suppl):2501–1. doi: 10.1200/jco.2005.23.16_suppl.2501

[40] Usyk M Pandey A Hayes RB Moran U Pavlick A Osman I. Bacteroides vulgatus and bacteroides dorei predict immune-related adverse events in immune checkpoint blockade treatment of metastatic melanoma. Genome Med (2021) 13(1):160. doi: 10.1186/s13073-021-00974-z 34641962 PMC8513370

[41] Omori M Maeda S Igarashi H Ohno K Sakai K Yonezawa T. Fecal microbiome in dogs with inflammatory bowel disease and intestinal lymphoma. J Vet Med Sci (2017) 79(11):1840–7. doi: 10.1292/jvms.17-0045 PMC5709562 28993566

[42] Marsella R. Advances in our understanding of canine atopic dermatitis. Vet Dermatol (2021) 32(6):547–e151. doi: 10.1111/vde.12965 33891338

[43] Gavazza A Rossi G Lubas G Cerquetella M Minamoto Y Suchodolski JS. Faecal microbiota in dogs with multicentric lymphoma. Vet Comp Oncol (2018) 16(1):E169–75. doi: 10.1111/vco.12367 29152844

[44] Pilla R Suchodolski JS. The role of the canine gut microbiome and metabolome in health and gastrointestinal disease . vol. 6, frontiers in veterinary science. Front Media SA (2020) 11. doi: 10.3389/fvets.2019.00498 PMC6971114 31993446

[45] You I Kim MJ. Comparison of gut microbiota of 96 healthy dogs by individual traits: Breed, age, and body condition score. Anim (Basel) (2021) 11(8):547–551. doi: 10.3390/ani11082432 PMC8388711 34438891

[46] Mrofchak R Madden C Evans MV Kisseberth WC Dhawan D Knapp DW. Urine and fecal microbiota in a canine model of bladder cancer. bioRxiv (2021) 16. doi: 10.1101/2021.12.20.472715.abstract

[47] Gilbert JA Blaser MJ Caporaso JG Jansson JK Lynch SV Knight R. Current understanding of the human microbiome. Nat Med (2018) 24(4):392–400. doi: 10.1038/nm.4517 29634682 PMC7043356

[48] Yatsunenko T Rey FE Manary MJ Trehan I Dominguez-Bello MG Contreras M. Human gut microbiome viewed across age and geography. Nature (2012) 486(7402):222–7. doi: 10.1038/nature11053 PMC3376388 22699611

[49] Huang Z Pan Z Yang R Bi Y Xiong X. The canine gastrointestinal microbiota: early studies and research frontiers. Gut Microbes (2020) 11(4):635–54. doi: 10.1080/19490976.2019.1704142 PMC7524387 31992112

[50] Peroni DG Nuzzi G Trambusti I Di Cicco ME Comberiati P. Microbiome composition and its impact on the development of allergic diseases. Front Immunol (2020) 11:700. doi: 10.3389/fimmu.2020.00700 32391012 PMC7191078

[51] Gasparrini AJ Crofts TS Gibson MK Tarr PI Warner BB Dantas G. Antibiotic perturbation of the preterm infant gut microbiome and resistome. Gut Microbes (2016) 7(5):443–9. doi: 10.1080/19490976.2016.1218584 PMC5154371 27472377

[52] van Nimwegen FA Penders J Stobberingh EE Postma DS Koppelman GH Kerkhof M. Mode and place of delivery, gastrointestinal microbiota, and their influence on asthma and atopy. J Allergy Clin Immunol (2011) 128(5):948–55.e1-3. doi: 10.1016/j.jaci.2011.07.027 21872915

[53] Burton EN O’Connor E Ericsson AC Franklin CL. Evaluation of fecal microbiota transfer as treatment for postweaning diarrhea in research-colony puppies. J Am Assoc Lab Anim Sci (2016) 55(5):582–7. https://www.ncbi.nlm.nih.gov/pubmed/27657714. PMC5029830 27657714

[54] Falony G Joossens M Vieira-Silva S Wang J Darzi Y Faust K. Population-level analysis of gut microbiome variation. Science. (2016) 352(6285):560–4. doi: 10.1126/science.aad3503 27126039

[55] Goodrich JK Waters JL Poole AC Sutter JL Koren O Blekhman R. Human genetics shape the gut microbiome. Cell (2014) 159(4):789–99. doi: 10.1016/j.cell.2014.09.053 PMC4255478 25417156

[56] Coelho LP Kultima JR Costea PI Fournier C Pan Y Czarnecki-Maulden G. Similarity of the dog and human gut microbiomes in gene content and response to diet. Microbiome (2018) 6(1):72. doi: 10.1186/s40168-018-0450-3 29669589 PMC5907387

[57] Ciaravolo S Martínez-López LM Allcock RJN Woodward AP Mansfield C. Longitudinal survey of fecal microbiota in healthy dogs administered a commercial probiotic. Front Vet Sci (2021) 8:664318. doi: 10.3389/fvets.2021.664318 34235200 PMC8255976

[58] Schmitz S Suchodolski J. Understanding the canine intestinal microbiota and its modification by pro-, pre- and synbiotics - what is the evidence? Vet Med Sci (2016) 2(2):71–94. doi: 10.1002/vms3.17 29067182 PMC5645859

[59] Ley RE Turnbaugh PJ Klein S Gordon JI. Human gut microbes associated with obesity. Nature (2006) 444(7122):1022–3. 10.1038/4441022a 17183309

[60] Tehrani AB Nezami BG Gewirtz A Srinivasan S. Obesity and its associated disease: a role for microbiota? Neurogastroenterol Motil (2012) 24(4):305–11. doi: 10.1111/j.1365-2982.2012.01895.x PMC3303978 22339979

[61] Salas-Mani A Jeusette I Castillo I Manuelian CL Lionnet C Iraculis N. Fecal microbiota composition changes after a BW loss diet in beagle dogs. J Anim Sci (2018) 96(8):3102–11. doi: 10.1093/jas/sky193 PMC6095273 29790949

[62] Vázquez-Baeza Y Hyde ER Suchodolski JS Knight R. Dog and human inflammatory bowel disease rely on overlapping yet distinct dysbiosis networks. Nat Microbiol (2016) 1:16177. doi: 10.1038/nmicrobiol.2016.177 27694806

[63] O’Keefe SJD. Diet, microorganisms and their metabolites, and colon cancer. Nat Rev Gastroenterol Hepatol (2016) 13(12):691–706. doi: 10.1038/nrgastro.2016.165 27848961 PMC6312102

[64] Ahn J Sinha R Pei Z Dominianni C Wu J Shi J. Human gut microbiome and risk for colorectal cancer. J Natl Cancer Inst (2013) 105(24):1907–11. doi: 10.1093/jnci/djt300 PMC3866154 24316595

[65] Roy S Trinchieri G. Microbiota: a key orchestrator of cancer therapy. Nat Rev Cancer (2017) 17(5):271–85. doi: 10.1038/nrc.2017.13 28303904

[66] Gur C Ibrahim Y Isaacson B Yamin R Abed J Gamliel M. Binding of the Fap2 protein of fusobacterium nucleatum to human inhibitory receptor TIGIT protects tumors from immune cell attack. Immunity (2015) 42(2):344–55. doi: 10.1016/j.immuni.2015.01.010 PMC4361732 25680274

[67] Chun JL Ji SY Lee SD Lee YK Kim B Kim KH. Difference of gut microbiota composition based on the body condition scores in dogs. Hanguk Tongmul Chawon Kwahakhoe Chi (2020) 62(2):239–46. doi: 10.5187/jast.2020.62.2.239 PMC7142278 32292931

[68] Song SJ Lauber C Costello EK Lozupone CA Humphrey G Berg-Lyons D. Cohabiting family members share microbiota with one another and with their dogs. Elife (2013) 2:e00458. doi: 10.7554/eLife.00458 23599893 PMC3628085

[69] Oh C Lee K Cheong Y Lee S-W Park S-Y Song C-S. Comparison of the oral microbiomes of canines and their owners using next-generation sequencing. PloS One (2015) 10(7):e0131468. doi: 10.1371/journal.pone.0131468 26134411 PMC4489859

[70] Mager LF Burkhard R Pett N Cooke NCA Brown K Ramay H. Microbiome-derived inosine modulates response to checkpoint inhibitor immunotherapy. Science (2020) 369(6510):1481–9. doi: 10.1126/science.abc3421 32792462

[71] Rooks MG Garrett WS. Gut microbiota, metabolites and host immunity. Nat Rev Immunol (2016) 16(6):341–52. doi: 10.1038/nri.2016.42 PMC5541232 27231050

[72] Canter RJ Grossenbacher SK Foltz JA Sturgill IR Park JS Luna JI. Radiotherapy enhances natural killer cell cytotoxicity and localization in pre-clinical canine sarcomas and first-in-dog clinical trial. J Immunother Cancer (2017) 5(1):98. doi: 10.1186/s40425-017-0305-7 29254507 PMC5735903

[73] Monjazeb AM Kent MS Grossenbacher SK Mall C Zamora AE Mirsoian A. Blocking indolamine-2,3-Dioxygenase rebound immune suppression boosts antitumor effects of radio-immunotherapy in murine models and spontaneous canine malignancies. Clin Cancer Res (2016) 22(17):4328–40. doi: 10.1158/1078-0432.CCR-15-3026 PMC5010514 26979392

[74] Tzeng A Sangwan N Jia M Liu C-C Keslar KS Downs-Kelly E. Human breast microbiome correlates with prognostic features and immunological signatures in breast cancer. Genome Med (2021) 13(1):1–17. doi: 10.1186/s13073-021-00874-2 33863341 PMC8052771

[75] Pushalkar S Hundeyin M Daley D Zambirinis CP Kurz E Mishra A. The pancreatic cancer microbiome promotes oncogenesis by induction of innate and adaptive immune suppression. Cancer Discovery (2018) 8(4):403–16. doi: 10.1158/2159-8290.CD-17-1134 PMC6225783 29567829

[76] Riquelme E Zhang Y Zhang L Montiel M Zoltan M Dong W. Tumor microbiome diversity and composition influence pancreatic cancer outcomes. Cell (2019) 178(4):795–806.e12. doi: 10.1016/j.cell.2019.07.008 31398337 PMC7288240

[77] Nejman D Livyatan I Fuks G Gavert N Zwang Y Geller LT. The human tumor microbiome is composed of tumor type-specific intracellular bacteria. Science (2020) 368(6494):973–80. doi: 10.1126/science.aay9189 PMC7757858 32467386

[78] Yust-Katz S Gigi E Rosenberg D Kanner AA Laviv Y Benouaich-Amiel A. Tami-40. tumor microbiome and glioblastoma (gbm). Neuro Oncol (2020) 22(Supplement_2):ii221–2.

[79] Zhu G Su H Johnson CH Khan SA Kluger H Lu L. Intratumour microbiome associated with the infiltration of cytotoxic CD8+ T cells and patient survival in cutaneous melanoma. Eur J Cancer (2021) 151:25–34. doi: 10.1016/j.ejca.2021.03.053 33962358 PMC8184628

[80] Azimi F Scolyer RA Rumcheva P Moncrieff M Murali R McCarthy SW. Tumor-infiltrating lymphocyte grade is an independent predictor of sentinel lymph node status and survival in patients with cutaneous melanoma. J Clin Oncol (2012) 30(21):2678–83. doi: 10.1200/JCO.2011.37.8539 22711850

[81] Loi S Drubay D Adams S Pruneri G Francis PA Lacroix-Triki M. Tumor-infiltrating lymphocytes and prognosis: A pooled individual patient analysis of early-stage triple-negative breast cancers. J Clin Orthod (2019) 37(7):559–69. doi: 10.1200/JCO.18.01010 PMC7010425 30650045

[82] Kobayashi N Hiraoka N Yamagami W Ojima H Kanai Y Kosuge T. FOXP3+ regulatory T cells affect the development and progression of hepatocarcinogenesis. Clin Cancer Res (2007) 13(3):902–11. doi: 10.1158/1078-0432.CCR-06-2363 17289884

[83] Zhai Y Haresi AJ Huang L Lang D. Differences in tumor initiation and progression of melanoma in the BrafCA ;Tyr-CreERT2;Ptenf/f model between male and female mice. Pigm Cell Melanoma Res (2020) 33(1):119–21. doi: 10.1111/pcmr.12821 PMC6928400 31449725

[84] Simpson S Dunning MD de Brot S Grau-Roma L Mongan NP Rutland CS. Comparative review of human and canine osteosarcoma: morphology, epidemiology, prognosis, treatment and genetics. Acta Vet Scand (2017) 59(1):71. doi: 10.1186/s13028-017-0341-9 29065898 PMC5655853

[85] Wunder JS Gokgoz N Parkes R Bull SB Eskandarian S Davis AM. TP53 mutations and outcome in osteosarcoma: a prospective, multicenter study. J Clin Oncol (2005) 23(7):1483–90. doi: 10.1200/JCO.2005.04.074 15735124

[86] Kirpensteijn J Kik M Teske E Rutteman GR. TP53 gene mutations in canine osteosarcoma. Vet Surg (2008) 37(5):454–60. doi: 10.1111/j.1532-950X.2008.00407.x 18986312

[87] Gardner HL Sivaprakasam K Briones N Zismann V Perdigones N Drenner K. Canine osteosarcoma genome sequencing identifies recurrent mutations in DMD and the histone methyltransferase gene SETD2. Commun Biol (2019) 2:266. doi: 10.1038/s42003-019-0487-2 31341965 PMC6642146

[88] Sakthikumar S Elvers I Kim J Arendt ML Thomas R Turner-Maier J. SETD2 is recurrently mutated in whole-exome sequenced canine osteosarcoma. Cancer Res (2018) 78(13):3421–31. doi: 10.1158/0008-5472.CAN-17-3558 29724721

[89] Amin SB Anderson KJ Boudreau CE Martinez-Ledesma E Kocakavuk E Johnson KC. Comparative molecular life history of spontaneous canine and human gliomas. Cancer Cell (2020) 37(2):243–57.e7. doi: 10.1016/j.ccell.2020.01.004 32049048 PMC7132629

[90] Alsaihati BA Ho K-L Watson J Feng Y Wang T Dobbin KK. Canine tumor mutational burden is correlated with TP53 mutation across tumor types and breeds. Nat Commun (2021) 12(1):4670. doi: 10.1038/s41467-021-24836-9 34344882 PMC8333103

[91] Gustafson DL Duval DL Regan DP Thamm DH. Canine sarcomas as a surrogate for the human disease. Pharmacol Ther (2018) 188:80–96. doi: 10.1016/j.pharmthera.2018.01.012 29378221 PMC6432917

[92] Siegel RL Miller KD Fuchs HE Jemal A. Cancer statistics, 2022. CA Cancer J Clin (2022) 72(1):7–33. doi: 10.3322/caac.21708 35020204

[93] Dobson JM. Breed-predispositions to cancer in pedigree dogs. ISRN Vet Sci (2013) 2013:941275. doi: 10.1155/2013/941275 23738139 PMC3658424

[94] Labadie J Swafford B DePena M Tietje K Page R Patterson-Kane J. Cohort profile: The golden retriever lifetime study (GRLS). PloS One (2022) 17(6):e0269425. doi: 10.1371/journal.pone.0269425 35679242 PMC9182714

